# Blueberry ripening mechanism: a systematic review of physiological and molecular evidence

**DOI:** 10.1093/hr/uhaf126

**Published:** 2025-05-14

**Authors:** Jose Martin Zapien-Macias, Tie Liu, Gerardo H Nunez

**Affiliations:** Horticultural Sciences Department, Institute of Food and Agricultural Sciences, University of Florida, PO Box 110690, Gainesville, FL, USA; Horticultural Sciences Department, Institute of Food and Agricultural Sciences, University of Florida, PO Box 110690, Gainesville, FL, USA; Horticultural Sciences Department, Institute of Food and Agricultural Sciences, University of Florida, PO Box 110690, Gainesville, FL, USA

## Abstract

Blueberry (*Vaccinium* spp. section Cyanococcus) ripening is a complex process involving physiological and molecular changes that affect harvest timing, fruit quality, and market value. This review examines scientific literature on blueberry ripening, aiming to establish a unified phenological framework for lowbush (*Vaccinium angustifolium*), highbush (*Vaccinium corymbosum*, including northern and southern types), and rabbiteye (*Vaccinium virgatum* Ait; syn. *Vaccinium ashei* Reade) blueberries. Blueberries follow a double-sigmoid growth pattern, with epidermis color changes marking the onset of ripening. Traditionally, fruits are classified as climacteric or nonclimacteric based on respiration rates and ethylene production. However, blueberry genotypes exhibit significant variability in these traits. Some genotypes exhibit high respiration rates during fruit color transition, but ethylene production maxima vary or may be absent. The diversity among blueberry genotypes and differences in research methodologies contribute to inconsistencies in reported data. Thus, a unified classification of blueberry ripening remains premature. Nevertheless, agronomic practices and ripening-related gene networks are available to enable future studies. This review also explores the implications of these findings for farmers and consumers.

## Introduction

Blueberries (*Vaccinium* spp. section cyanococcus) are a popular fruit due to the health benefits associated with consumption of their anthocyanins [1, 2]. Anthocyanins are visible flavonoids in the skin of blueberries that impart their characteristic red and blue colors, serving as indicators of fruit ripeness [3, 4]. The process of ripening affects fruit firmness, soluble solids concentration, titratable acidity, and volatiles profile [5]. Thus, harvesting fruits at their peak ripeness is essential to meet consumer expectations for quality, taste, and potential health benefits.

Fruit ripening is a complex mechanism that involves biochemical, metabolic, and molecular changes affecting fruit appearance (e.g. color) and organoleptic attributes (e.g. texture, flavor, and aroma) [6]. Biochemical changes include chlorophyll catabolism, carotenoid and/or flavonoid synthesis, cell wall disassembly, accumulation of sugars and volatile organic compounds, and decline in organic acids [6, 7]. Metabolic changes include increases in fruit respiration rates, phytohormone metabolism, and shifts in starch and organic acid metabolism. These changes are regulated by complex gene networks (e.g. ripening-specific gene regulation) and protein accumulation patterns [6]. While these mechanisms have been studied across different blueberry genotypes and species, varying results limit a comprehensive understanding of blueberry ripening. This review aims to synthesize the available evidence and present a unified framework for future studies focused on blueberry ripening.

## Fruit ripening mechanism

Fruit ripening is traditionally classified based on the presence (climacteric, CL) or absence (nonclimacteric, NC) of a cellular respiration peak accompanied by a preceding, concurrent, or subsequent increase in ethylene biosynthesis [8–10]. Ethylene is the main hormone regulator during CL ripening. The precursor for ethylene synthesis is 1-aminocyclopropane-1-carboxylic acid (ACC). ACC is synthesized by ACC synthase (ACS), which is the rate-limiting enzyme in this process. ACC is subsequently transformed into ethylene by ACC oxidase (ACO) [11]. ACS and ACO are regulated by two distinct systems that control ethylene production. In System 1, the activity of ACS is typically autoinhibited (ethylene concentration in the tissue inhibits additional ACC synthesis). Environmental and endogenous factors such as wounding, drought, flooding, auxin, etc., can activate ACS for ethylene production during the normal growth and development [11, 12]. In contrast, System 2 entails autocatalytic ethylene production (ethylene concentration in the tissue promotes additional ACS and ACO activity) [12]. System 2 is involved in CL ripening, organ senescence, or in response to exogenous ethylene applications. In this system, increasing ethylene levels affect CL fruit physical and chemical characteristics.

**Figure 1:**
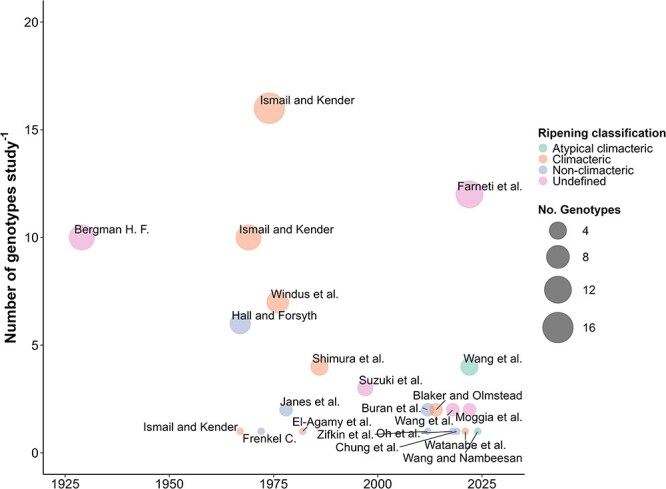
Blueberry fruit ripening studies by year of publication. Colored dots indicate the ripening classification, while dot size indicates the number of genotypes used in each study.

NC ripening has been hypothesized to be ethylene-independent [13] and mostly characterized by a gradual decrease in respiration as ripening progresses [14]. However, recent studies have highlighted the role of ethylene biosynthesis and signaling pathway in NC fruits [15]. For instance, trace amounts of endogenous ethylene have been described at the onset of NC fruits like grape (*Vitis vinifera* L.). This ethylene is thought to play a critical role in enhancing fruit size, reducing fruit acidity, and inducing the accumulation of abscisic acid (ABA) and anthocyanins [16, 17].

The CL ripening process has been thoroughly studied in tomato (*Solanum lycopersicum*) and apple (*Malus domestica*) [18], while the NC ripening process has been mainly described in strawberry (*Fragaria* × *ananassa*) and grape [19, 20]. Although strawberry is a globally important commodity, the extrapolation of this model to other NC fruit species has limitations due to morphological differences [21]. On the other hand, grape might be a more suitable model for comparison with blueberry. Grapes and blueberries exhibit a double sigmoid growth curve and undergo color transitions that mark distinct ripening stages [20]. However, significant differences are observed in the temporal patterns of ethylene production and respiration rates among these crops [20, 22]. These differences make it challenging to directly apply grape ripening models to blueberry and they emphasize the need for crop-specific research.

## Bibliographic search about blueberry ripening

Numerous studies focusing on the ripening of blueberry fruits have been published over the last century (Fig. 1). A bibliographic search in Clarivate’s Web of Science using keywords ‘blueberry’ and ‘ripening’ in the fields [Topic], and [Article Title], respectively, yielded 85 primary research articles. The oldest study was conducted in 1929 and the most recent in 2024. We narrowed down our bibliographic search to papers featuring keywords such as ‘climacteric’, ‘nonclimacteric’, ‘ethylene’, and ‘respiration’ according to abstract and title relevance. Full texts of the selected papers were then downloaded and evaluated according to their relevance in determining the respiration rate and ethylene evolution as these are important characteristics in the classification of ripening mechanism [14]. Each of these studies employed different methodologies and genotypes to explore the blueberry ripening mechanism, which is further described in the following sections.

## General features of blueberry fruit development and ripening

Domestic blueberries display diverse morphologies, ploidy levels, and interspecific hybridization [23]. Blueberry fruits can have numerous seeds or be seedless due to parthenocarpy [24]. The fruit ripens over a span of 2–3 months after pollination depending on the cultivar and environmental conditions [25]. Highbush blueberry (*Vaccinium corymbosum* L.), rabbiteye blueberry (*Vaccinium virgatum* Ait; syn. *Vaccinium ashei* Reade), and lowbush blueberry (*Vaccinium angustifolium* Ait.) are the predominant cultivated types in the blueberry industry [26]. Highbush genotypes are further categorized into northern (NHB) and southern (SHB, *V. corymbosum* L. interspecific hybrids) upon their chilling requirements [26]. NHB and lowbush (LB) blueberries exhibit the highest chilling requirement (>800 h), followed by rabbiteye (RE; 450–500 h), and SHB (0–100 h) [26].

**Figure 2:**
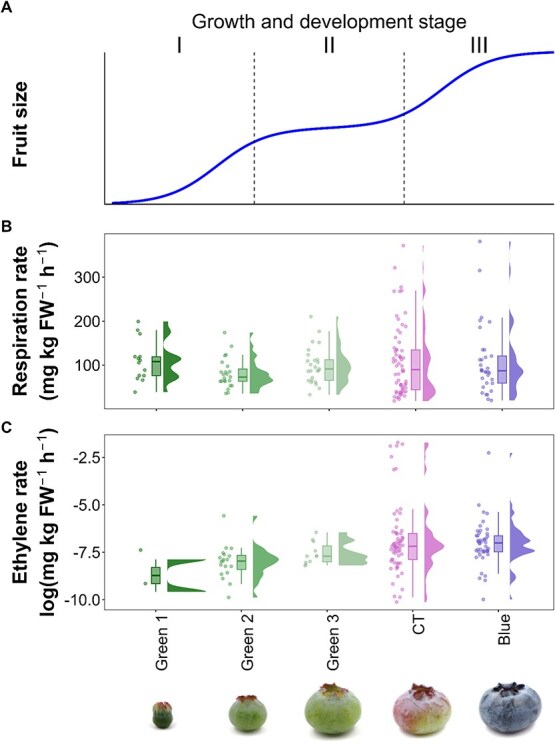
Double sigmoid blueberry fruit growth (A), fruit respiration rate (B, *n* = 185), and ethylene production rate (C, *n* = 212) across various stages of fruit development (x-axis). Data for panels B and C were compiled from the literature. Ethylene data are log-transformed to facilitate visualization. FW = fresh weight.

Overall, blueberry fruit development follows a double sigmoid curve divided into three stages [25, 27]. Stage I starts after pollination, and it is characterized by growth due to cell division [27]. Stage II is characterized by stagnated growth, at a time when seed and embryo development takes place (Fig. 2A). Increasing metabolism of organic acids and other defense and stress resistance compounds (e.g. proanthocyanidins and flavonols) have been reported at the early stages of development when the fruit is formed and remains green [4, 28]. Stage III marks the resumption in growth followed by ripening, which is characterized by changes in fruit epidermis color as well as respiration rate, and ethylene production (Fig. 2B and C). This stage is associated with the attainment of horticultural maturity, which refers to a fruit that has achieved optimal organoleptic quality, including cell wall disassembly, textural changes, regulation of organic acids, and increases in soluble sugars and aroma volatile production [29].

Blueberries display asynchronous ripening. Fruits on the same cane, branch, and inflorescence ripen at different times and rates, resulting in extended harvesting periods. Fruit development varies among blueberry genotypes and is influenced by factors such as crop load, chilling hour accumulation, and growing degree days accumulation [30, 31]. However, blueberry ripening can be also affected by farming practices and management. For instance, the use of bees (*Apis mellifera* L.) for cross-pollination increases the proportion of early-ripening fruit [32]. Also, cultivation in high tunnels, which elevate daytime air temperatures, can shorten the interval from bloom to harvest [33]. The application of plant growth regulators can accelerate fruit ripening by increasing the proportion of ripe fruit in each harvest [34, 35]. Conversely, blueberry plants managed under an evergreen system, where dormancy is avoided and foliage is retained year-round, typically display a protracted harvest period due to diversity in fruit development start points and ripening rates. Long ripening periods often require multiple harvests, which negatively impact labor cost and grower profitability. Inconsistencies and knowledge gaps about blueberry fruit ripening hinder the blueberry industry because harvest operations, postharvest quality, and ultimately consumer experiences depend directly on practices aimed at harvesting these fruits at an optimum time.

In this review, we summarize the key findings across physiological, biochemical, hormonal, and molecular aspects of fruit ripening across the predominant cultivated blueberry species and types. Additionally, we identified common elements across various blueberry ripening studies, including fruit development scales, color change and its relationship with ripening, fruit respiration rates in association with ethylene production, etc. These insights offer opportunities to evaluate the various ripening classifications proposed for blueberry over nearly a century of research. Accurate identification and description of blueberry ripening is essential for developing agronomic and breeding strategies that support the blueberry industry. These strategies could enhance harvest efficiency, postharvest handling and storage, improve disease resistance, and enhance fruit flavor and nutritional content.

## Color change and its association with blueberry ripening

Horticultural crops must be harvested at a maturity stage that achieves consumer satisfaction and repeated purchases [29]. Fruit color correlates with blueberry ripening, and, therefore, it is used to determine optimal time for harvesting [36]. Blueberry fruits turn from green to blue due to anthocyanin accumulation. Five main anthocyanidins have been described in blueberry fruits: cyanidin, delphinidin, peonidin, petunidin, and malvidin [4, 37, 38]. Cyanidin is usually present at early stages of development (from green 1 to 3), while the rest accumulate from the ‘color transition’ to the ‘blue’ stage (Fig. 2). In this review, we refer to ‘color transition’ as the fruit development stage that coincides with changes in fruit skin color as a consequence of chlorophyll breakdown and anthocyanin accumulation [4, 37]. Anthocyanin accumulation in blueberries is strongly regulated by development, temperature, light intensity/quality, genotype, and species [39–42]. Warmer temperatures and higher solar radiation usually result in accelerated fruit color changes [39]. Anthocyanin accumulation is a photoprotective mechanism in the fruit epidermal cells against ultraviolet and blue light [4, 39]. Rabbiteye and some NHB fruits present a distinct color transition stage, characterized by prolonged and uniform pink hues in the fruit epidermis before turning fully blue. In contrast, SHB genotypes display a transient and uneven color transition stage with fruits having a mosaic of green, pink, and blue hues at the same time. These differences in coloration patterns may be linked to variations in anthocyanin concentration at different developmental stages among blueberry types and species [4, 28].

**Table 1:** Ripening classifications based on the color transition stage across several blueberry ripening studies.

Ripening class	Identifier	Description	Reference
Mature green	MG	Visual signs of chlorophyll breakdown; pale green color around the calyx	[3, 36, 43–46]
Breaker	BR	First signs of color change (green to pink-red)	[47]
Green-pink	GP	Pale green with traces of pink coloration, especially around the calyx end	[3, 36, 44, 45]
Semi-red	SR	Mostly green color with blush patches	[48]
Red	S6	50% red skin	[4, 49]
Pink	P	50% pink to 100% pink color	[9, 34, 43, 48, 49]
Pink-red	PR	Largely pink color, very little if any green; red coloration at the calyx end	[3, 39]
Pink-purple	PP	100% pink + purple colors	[50]
Red-purple	S7; RP	Predominantly purple skin with some red or blue; reddish purple	[4, 37, 41]
Blue-pink	BP	Mostly blue with pink colors at the stem end	[36, 44, 45]
Red-blue	RB	Reddish color with dark red and blue colors; red coloration evident at the calyx end	[3]

Across the literature, authors have used a variety of scales to categorize blueberry fruit development. Scales used range from 3 to 8 classes and most of them are based on fruit size and color. The selection of a scale is generally influenced by the specific objectives and/or blueberry types and species used in the study. For instance, researchers have used different scales to assess respiration and ethylene production during fruit development of LB, NHB, SHB, and RE genotypes [3, 9, 36]. Discrepancies in scales have been also observed between studies that focus on the same blueberry species and share similar research objectives [9, 36]. Within these scales, green and blue stages are clearly identified in most ripening scales. However, the categorization of the color transition stage remains nuanced, resulting in various nomenclatures in different studies. Most of these nomenclatures can be represented with the term ‘color transition’ as shown in Table 1. To date, a standardized scale has not been universally adopted in blueberry fruit development studies. In this review, we use the development stages detailed in Fig. 2 to facilitate discussion.

The color transition stage marks the onset of blueberry fruit ripening (Fig. 2). This stage is characterized by chlorophyll catabolism and the accumulation of anthocyanins and other pigments [37, 51]. Many ripening studies have utilized scales that subdivide the color transition stage into narrower, temporally distinct classes (e.g. MG, GP, BP; Table 1), providing finer detail on the progression of color changes during ripening [3, 36]. These studies have reported significant differences in fruit respiration between these substages. Similarly, other studies assessing ethylene production, and carbohydrate and organic acids concentrations have also described significant differences when the color transition stage was divided into more distinct categories [28, 43, 47]. This suggests that developmental scales can be tailored to align with the specific traits of interest and the objectives of the study. Although not explicitly stated by the authors, the use of varying scales may reflect an effort to capture as much detail as possible about traits of interest throughout the continuous progression of fruit color changes.

## Respiration rate and ethylene evolution in blueberries

Fruit respiration during ripening is the process that supplies energetic resources for other ripening events such as protein and pigment synthesis [14]. The first blueberry ripening studies quantified fruit respiration rates (measured as CO_2_ production or O_2_ consumption) across multiple NHB genotypes [52]. The most striking finding was that the greatest respiration rate occurred during the early color transition stage. Additionally, respiration rates decreased as fruit advanced toward the fully ripe stage (blue fruit). These observations suggested that NHB fruits exhibit CL ripening. Notably, genotypes that exhibited lower respiration rates (e.g. ‘Rubel’) were found to maintain better postharvest fruit quality than genotypes with higher respiration rates [52]. An interest in improving blueberry shelf life and quality resulted in further fruit respiration studies using LB genotypes [53]. Hall and Forsyth [50] considered LB blueberries as NC because tested fruits exhibited low respiration rates during ripening. On the other hand, Ismail and Kender [3] noted a respiration peak during the color transition stage in both NHB and LB blueberry fruits. Eventually, the documented fruit respiration rates led to the conclusion that blueberries followed a CL ripening mechanism. Later, Windus *et al*. [36] and Suzuki *et al*. [45] studied the respiration rate and ethylene evolution of SHB. These studies described peak respiration rates during the color transition stage, reinforcing the notion that blueberries are CL fruit. These findings were also supported by subsequent studies [43, 44].

The evaluation of fruit respiration based on the progression of fruit color is a common denominator among these studies [3, 9, 36, 43–45, 50]. The studies compiled in Fig. 2 indicate that respiration rates in blueberry fruit vary continuously throughout fruit development. These fluctuations in respiration may indicate physiological changes occurring in the fruit tissues, such as ethylene production, shifts in metabolic pathways, or accelerated ripening during development [14]. Despite the observed variability in the dataset, maximum respiration rates were heterogenous and highly variable among the tested genotypes. The highest respiration rate magnitudes were generally observed during the color transition and blue stages, as expected during climacteric ripening (Fig. 2B).

Ethylene evolution is another element in CL ripening. Blueberry ethylene evolution has been previously studied [9, 36, 43–45, 47, 48]. Frenkel [48] used a four-stage developmental scale and did not observe an ethylene production maximum over the fruit development. Subsequent studies employed different fruit development scales (including 3–6 stages) and detected ethylene production maxima, albeit at different stages [9, 36, 44, 45]. Additionally, Wang *et al*. [9] and Shimura *et al*. [44] reported distinct ethylene concentrations across different blueberry species and among genotypes within the same species. Blueberries exhibit a marked increase in their ethylene production rate during fruit development (Fig. 2C), consistent with observations reported by other researchers [54]. Maximum ethylene production levels are typically observed in the color transition and blue stages.

Traditionally, the presence of ethylene and fruit respiration maxima has been employed for the classification of fruits as CL or NC [6, 14]. Data on respiration and ethylene levels during fruit development exhibit variability across different blueberry species and between genotypes of the same species (Supplementary Fig. S1). However, the color transition stage seems to be the converging point for both respiration (32.4–395.1 mg kg^−1^ h^−1^) and ethylene (0.00005–7.0 mg kg^−1^ h^−1^) maxima (Supplementary Table S1). Respiration maxima observed in RE and SHB are higher than those observed in other NC crops like grapes, strawberries and cherries (*Prunus avium* L.) and even exceed those of some CL crops such as tomatoes and apples [14, 55]. Respiration rates peak in SHB and LB during the color transition stage, whereas in NHB respiration peaks occur during the color transition and blue stage. Notably, RE blueberries exhibit respiration maxima in each stage of fruit development (Supplementary Fig. S1C). Diverse respiration rates are associated to varying cellular metabolic rates [56], seasonal weather variations, the timing of fruit sample collection, and the developmental stage of the fruit [36]. Nonetheless, the variability in the magnitude of fruit respiration among blueberry species remains largely unexplained. On the other hand, RE genotypes exhibited higher ethylene production maxima than NHB and SHB genotypes (Supplementary Fig. S1F–H). These observations are consistent with recent studies showing that ethylene production in NHB, SHB, and RE genotypes vary depending on both the cultivar and species [9, 47]. Of the studies examined, 88% reported ethylene evolution rates between 0.00005 and 0.22 mg kg^−1^ h^−1^, while the remainder reported much higher levels between 2000 and 7000 mg kg^−1^ h^−1^. These ethylene measurements were conducted using gas chromatography equipment. However, there were differences in sample manipulation and analysis between these studies (Supplementary Table S1). Storage temperature and measurement timing significantly affect ethylene production in blueberries [57]. High variability in reported ethylene levels makes results interpretation and meta-analysis challenging. Standardized methods are essential for accurately measuring ethylene production during blueberry ripening. The scientific community is encouraged to adopt a standardized method for this kind of analysis.

On the basis of the evidence available to date, blueberries appear to exhibit ethylene production and respiration maxima at different stages of development (most commonly during the color transition stage), marking the onset of ripening.

## Genetic regulation of blueberry ripening

**Figure 3:**
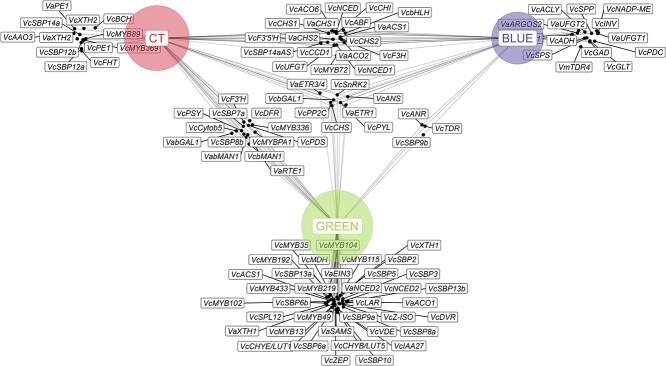
Gene interaction network across blueberry fruit development stages. Data for the gene interaction network were sourced from the literature. Green, color transition (CT), and blue stages are represented by nodes. The size of each stage node is proportional to the number of associated genes (black dots).

Advancements in molecular techniques have improved our understanding of the biosynthetic pathways involved in blueberry fruit development. In this review, we compiled 94 differentially expressed (DE) genes from various studies across various stages of fruit development. Then, we grouped DE genes using the previously described development scale (Supplementary Table S2): 57 genes were DE during the green stage, 45 during the color transition stage, and 41 during the blue stage (Fig. 3). Some of the DE genes function exclusively during one developmental stage, while others are involved in multiple stages. The description of blueberry development in Fig. 2 was employed to illustrate the molecular mechanisms impacting fruit esthetic and organoleptic attributes. Further details can be also found in Supplementary Table S2.

During the green stage, DE genes are involved in fruit growth, fruit color, and ethylene production. Differential expression at this stage was typically assessed by comparing transcript abundance in the green stage to levels immediately after petal fall. The control of fruit size is influenced by the transcript abundance of an *Aux/IAA* gene (*VcIAA27*) [58]. Additionally, high transcript abundance of *XYLOGLUCAN ENDOTRANSGLUCOSYLASE/HYDROLASE1* (*VcXTH1*, *VaXTH1*), *XYLOGLUCAN ENDOTRANSGLUCOSYLASE/HYDROLASE2* (*VcXTH2*, *VaXTH2*), *1*,*4-β-MANNOSIDASE1* (*VcbMAN1*, *VabMAN1*), *PECTINESTERASE1* (*VcPE1*, *VaPE1*), and *β-GALACTOSIDASE1* (*VcbGAL1*, *VabGAL1*) are observed at this stage [59]. These genes encode cell wall breakdown enzymes, which are involved in cell expansion. Different members of the same gene family for the same enzymes may play a role in cell wall expansion and fruit softening. Additionally, these enzymes have been also reported for fruit softening in other crops, such as persimmons (*Diospyros kaki L.)* and strawberries [60, 61].

The characteristic green skin color, caused by high chlorophyll and low anthocyanin accumulation, has been linked to the high expression of transcription factors from the *SQUAMOSA Promoter Binding Protein* (*VcSBP*) family (*VcSBP2*, *VcSBP3*, *VcSBP5*, *VcSBP6a*, *VcSBP6b*, *VcSBP8a*, *VcSBP9a*, *VcSBP10*, *VcSBP13a*, and *VcSBP13b*) [62] and genes from the R2R3-MYB group (*VcMYB433*, *VcMYB102*, *VcMYB336*, *VcMYB192*, *VcMYB35*, *VcMYB104*, *VcMYB219*, *VcMYB115*, *VcMYB49*, *and VcMYB13*) [63]. In addition, expression of genes in the R2R3-MYB group is associated with proanthocyanidin production, which function as herbivore repellents in the green fruit [4]. Also, there is active negative regulation of the ethylene biosynthetic pathway. *SQUAMOSA PROMOTER-BINDING PROTEINLIKE12* (*VcSPL12*) genes inhibit the transcription of ethylene biosynthetic genes *ACC SYNTHASE1* (*VcACS1*) and *ACC OXYGENASE6* (*VcACO6*)*.* Ethylene synthesis early in the fruit development is linked to System 1 ethylene. Increased expression of *S-ADENOSYLMETHIONINE SYNTHASE* (*VaSAMS*) was observed at the early green stages of RE genotypes and decreased with ripening progression [64]. High expression of *VcSPL12* maintains basal ethylene production in NHB, while simultaneously suppressing anthocyanin biosynthesis and contributing to increased chlorophyll levels. [65]. *ACS1* is the gene encoding ACS, the rate-limiting enzyme in the ethylene biosynthesis pathway in RE [9] and NHB [65]. In RE, *VaACS1* expression was higher during the blue stage compared to early green fruit and color transition stages. However, this effect appears to be cultivar-dependent, as it was not consistently observed across all evaluated genotypes. In contrast, in NHB blueberries, *VcACS1* expression remained constant across all fruit developmental stages. Exogenous applications of ethephon to RE genotypes resulted in a temporary reduction in *VaACS1* transcript abundance, along with a decrease in ACC concentration. These findings suggest the absence of an autocatalytic System 2 ethylene response at the level of ACC synthesis [9]. It follows that ACS enzymatic activity during blueberry ripening may be minimal and negatively affected by exogenous ethylene. While the autocatalytic ethylene cycle appears to be absent in some blueberry types, low but consistent ethylene concentrations have been reported in several genotypes (Fig. 3).

The final enzymatic step of the ethylene biosynthesis pathway is catalyzed by ACO, an enzyme encoded by *VaACO1*, *VaACO2*, and *VcACO6*. In RE blueberries, *VaACO1* was overexpressed during the early green stages [9], while *VaACO2* and *VcACO6* displayed increased expression at the later stages of fruit development in RE and NHB, respectively [9, 65]. Unlike the response of *ACS* transcripts to exogenous ethephon applications, *ACO1* and *ACO2* levels were enhanced by ethephon in RE genotypes, indicating that there is a functional autocatalytic response downstream from *ACS* [9]. Notably, *ACO* genes exhibited high expression levels at the onset of ripening, even when *ACS* gene expression was low. This has also been reported in NC crops like strawberries [66] and grapes [16], where a slight increase in endogenous ethylene at the onset of ripening was sufficient to elevate *ACO* gene expression. The importance of the coordination of expression and activity between ACS and ACO for NC ripening remains an area for further investigation.

Most of these studies focused on *V. corymbosum* genotypes, while a few also included *V. virgatum* (syn. *V. ashei*) and *Vaccinium myrtillus*. There seems to be different homologs of the same gene in other blueberry species, such as *VaACO2* (in *V. ashei*) and *VcACO6* (in *V. corymbosum*) (Supplementary Table S1). Also, during the CL ripening of tomato, the expression of *SlACS1* and *SlACS6* maintain ethylene production at basal levels, while *SlACS2* and *SlACS4* are overexpressed during the transition from System 1 to System 2 of ethylene production at the onset of ripening [67]. In NHB blueberries, Li *et al*. [65] did not detect expression of *VcACS4* or other members of the ethylene biosynthesis or signaling pathways. Alternatively, in citrus, *CsACS1* and *CsACS2* regulate ethylene synthesis before fruit coloring, while stable and low levels of endogenous ethylene synthesis during ripening are maintained through the expression of *CsACS3*, *CsACS4*, and *CsACS6*.

Further, key components of ethylene signaling pathways are *AUXIN-REGULATED GENE INVOLVED IN ORGAN SIZE* (*VaARGOS2*), *REVERSION TO ETHYLENE SENSITIVITY1* (*VaRTE1*), *ETHYLENE INSENSITIVE3* (*VaEIN3*), and *ETHYLENE RECEPTOR3/4* (*VaETR3/4*). Their expression is generally upregulated between the color transition and the blue stage of ripening [9, 64], but their role in physiological responses is less well understood.

Recent studies have confirmed that ethylene metabolism and signaling coordinate color change through chlorophyll and carotenoid catabolism, disassembly of the photosynthesis apparatus, and increases in anthocyanin and ABA synthesis in blueberry [51, 59, 65]. In other crops, such as grapes, trace amounts of ethylene are necessary to activate other genes, like *VcNCED* [17]. In NHB blueberries, elevated ethylene levels coincided with the overexpression of *VcNCED1* [4]. These genes produce the enzyme NCED, which plays a crucial role in the ABA biosynthetic pathway and triggers the color transition during ripening [4, 17].

**Figure 4:**
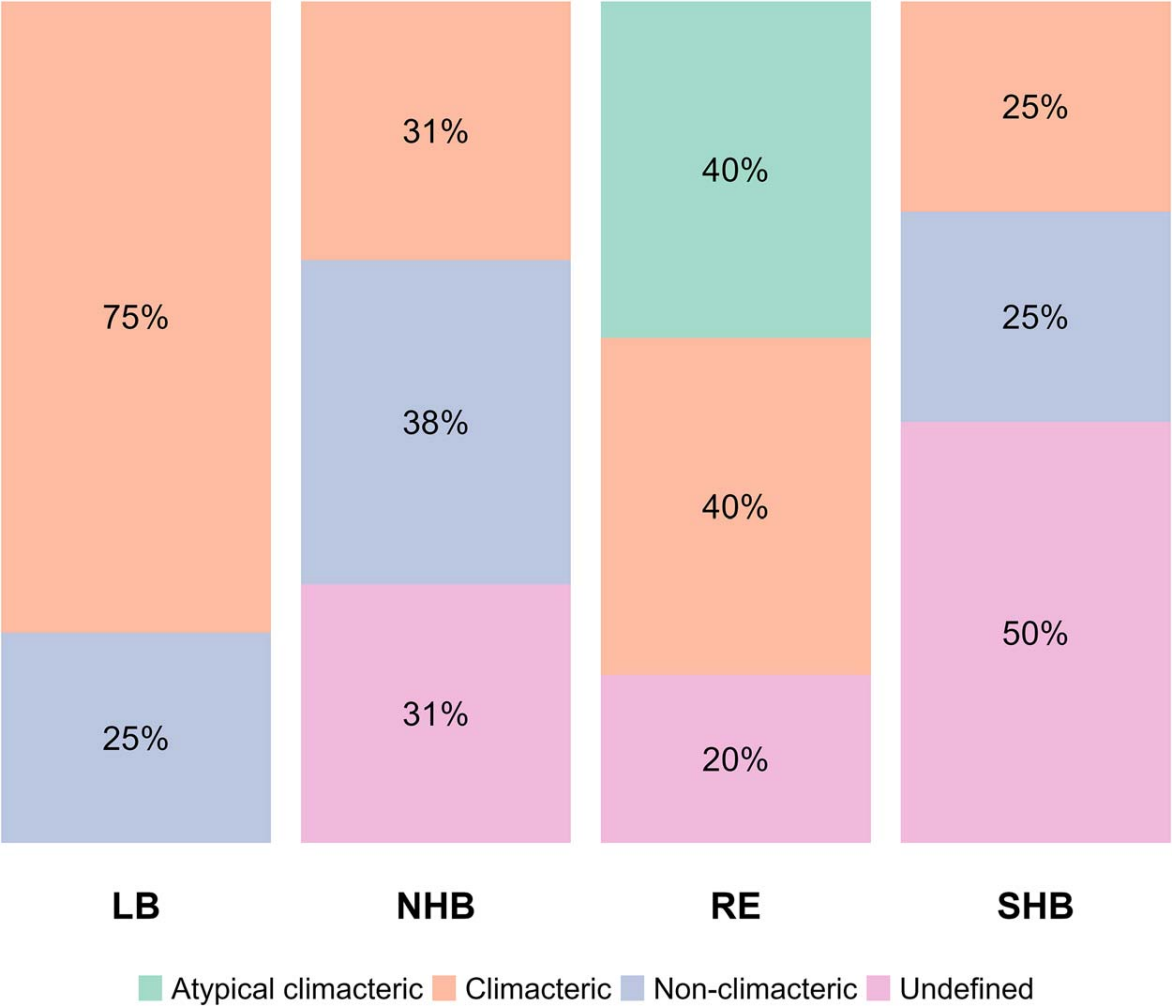
Fruit ripening classification across different types of blueberries. Lowbush blueberries (LB, *n* = 4 studies); northern highbush (NHB, *n* = 13); rabbiteye (RE, *n* = 5); and southern highbush (SHB, *n* = 4).

Several genes are DE during the color transition stage. Differential expression at this stage was typically assessed by comparing transcript abundance in the color transition stage to levels during the green stage. During the color transition stage, there was a decrease in the expression of transcripts associated with carotene biosynthesis, such as *PHYTOENE SYNTHASE* (*VcPSY*), *PHYTOENE DESATURASE* (*VcPDS*), and *ζ-CAROTENE ISOMERASE* (*VcZ-ISO*). Similarly, xanthophyll-related transcripts are reduced, including *ε-HYDROXYLASE/CAROTENE ε-MONOOXYGENASE* (*VcCHYE/LUT1*), *β-CAROTENE HYDROXYLASE/LUTEIN DEFICIENT 5* (*VcCHYB/LUT5*), *ZEAXANTHIN* (*VcZEP*), and *VIOLXANTHIN DE-EPOXIDASE* (*VcVDE*) [51]. Notably, carotenoids serve as precursors to a diverse array of apocarotenoids, some of which are further metabolized into important hormones, such as ABA [68]. A high concentration of ABA at the onset of ripening has been associated with regulation of anthocyanin biosynthesis [4, 69]. *CHALCONE SYNTHASE* (*VcCHS*) and *FLAVANONE-3β-HYDROXYLASE* (*VcFHT*), C*HALCONE ISOMERASE* (*VcCHI*), *DIHYDROFLAVONOL 4-REDUCTASE* (*VcDFR*), *FLAVONOID 3′ - HYDROXYLASE* (*VcF3’H*), *UDP-GLUCOSE: FLAVONOID-3-O-GLYCOSYLTRANSFERASE* (*VcUFGT*), *ANTHOCYANIDIN SYNTHASE* (*VcANS*), *FLAVANONE 3-HYDROXYLASE* (*VcF3H*), and genes from the subgroup-6 R2R3MYB protein (*VcMYBA*) exhibit a substantial increase as the fruit ripens [4, 69, 70]. These genes encode enzymes in the flavonoid pathway, essential for the production of proanthocyanidins, anthocyanins, and flavonols [4]. These expression patterns are consistent with the accumulation of anthocyanins leading to red and blue coloration of blueberries [4, 69].

Finally, several genes were also DE at the blue stage. Differential expression at this stage was typically assessed by comparing transcript abundance in the blube stage to levels during the color transition stage. Several transcription factors were DE to regulate sugar accumulation and organic acid catabolism. For instance, increased expression of *SUCROSE*-*PHOSPHATASE* (*VcSPP*), *SUCROSE*-*PHOSPHATE SYNTHASE* (*VcSPS*), and *INVERTASE* (*VcINV*) have been associated with higher sugar content (mainly glucose and fructose) in the fruit [71]. Genes involved in citrate [e.g. *ATP-CITRATE LYASE* (*VcACLY*), *GLUTAMATE DECARBOXYLASE* (*VcGAD*), and *GLUTAMATE SYNTHASE* (*VcGLT*)], malate [e.g. *ALCOHOL DEHYDROGENASE* (*VcADH*), *PYRUVATE DECARBOXYLASE* (*VcPDC*), and *NICOTINAMIDE ADENINE DINUCLEOTIDE PHOSPHATE-DEPENDENT MALIC ENZYME* (*VcNADP-ME*)] degradation, which ultimately contribute to flavor development [71] are also DE in these stages.

## Ripening classification of blueberries

Most studies presented in this review have focused on respiration and ethylene production to classify the ripening mechanism of blueberries. However, this approach has led to conflicting conclusions, resulting in a lack of consensus regarding the ripening classification of blueberries across different types and species (Fig. 4).

The original criterion used to classify blueberry ripening was fruit respiration rate increases during the CT stage [3, 52]. However, increases in respiration may not always indicate CL ripening mechanism. High respiration rates can represent a general physiological response to ethylene, whether produced internally (as in CL fruits) or exogenously applied. Both CL and NC fruits exhibit this response [14]. In fact, recent studies suggest that ethylene biosynthesis and signaling pathway elements are common in CL and NC fruits [15]. Ethylene generated during blueberry ripening appears to be produced by small amounts of ACC synthesized during the color transition stage [9, 45]. Even small amounts of ethylene (e.g. 1 μl l^−1^) might be sufficient to regulate the progression of blueberry ripening [9, 10]. Ethylene concentrations vary between CL and NC fruits [15]. Nonetheless, the magnitude of ethylene production can be misleading, as the critical factor to consider is when the tissue becomes more sensitive to ethylene and if the internal concentration reaches the threshold necessary to trigger biological responses [54, 72].

Ethylene concentration maxima have generally been observed during the color transition CT stage. However, most ripening-related traits (anthocyanin, sugars, and organic acid accumulation) continue to develop in the fruit even as ethylene levels decline [9, 28]. Studies using RE, SHB, and NHB genotypes have reported declining ethylene concentrations alongside decreased concentrations of ACC as ripening progresses toward the blue fruit stage [9, 45]. Some SHB genotypes also exhibit ethylene production during the late stages of ripening, with variations observed across different years of evaluation [9]. Further, there may be temporal and environmental factors influencing the activation of this system [9]. Wang *et al*. [9] observed that RE blueberries exhibit higher respiration rates during ripening but did not display autocatalytic System 2 ethylene. This conclusion was based on the absence of enhanced ACC concentrations and *ACS*-related gene expression following exogenous ethephon applications. These findings led to the classification of RE blueberries as atypical climacteric fruits (Fig. 4). Other fruit crops, like kiwifruit (*Actinidia chinensis*) and melon (*Cucumis melo* L.), exhibit atypical climacteric ripening [73–75] as they have ripening-associated traits that are independent of ethylene concentrations. Melon is a notable example, as this fruit crop comprises CL genotypes (e.g. *C. melo* var. *cantalupensis*) with high ethylene production, fast ripening rate, and short shelf life, and NC genotypes (e.g. *C. melo* var *inodorus*) that do not produce autocatalytic ethylene and generally exhibit a slower ripening rate but extended shelf life [75]. Whether all blueberry types and species share the same ripening classification remains an area for further investigation.

## Management practices to enhance blueberry ripening

Commercially, the timing of blueberry harvest depends on the coloration of fruit skin [35]. This approach has many limitations, as blueberries do not ripen synchronously on the plant. Thus, management practices that enhance fruit coloration, such as using harvest timing, plant growth regulator (PGR) application, and crop load management, have been previously used. Harvesting at early stages of ripeness can improve postharvest shelf life, although it may negatively affect flavor [25, 76]. Conversely, extended time on the bush leads to increased pigments, sugars, and phenolic compounds but reduces firmness [77]. Exogenous applications of ethephon testing different rates (200–8000 mg l^−1^) have been performed to accelerate blueberry ripening [34, 35, 78, 79]. Ethephon applications accelerated anthocyanin accumulation, reduced titratable acidity, and promoted fruit softening. These changes occurred at a faster pace than sugar accumulation [35]. However, caution is advised when using ethephon, as it has been reported to cause fruit abscission when applied at rates >2000 mg l^−1^ during early stages of fruit development (<25 days after full bloom) [79]. Additionally, its effects have shown variability over time, even when the same application rates were used [35].

Melatonin (300 mg l^−1^) and methyl jasmonate (10 mg l^−1^) have been used to increase soluble sugars in NHB blueberry [80]. Similarly, 1-methylcyclopropene (160 mg l^−1^) has been tested to improve fruit firmness in SHB [81]. Exogenous applications of ABA (600–1000 mg l^−1^) increased the proportion of color transition fruit due to accumulation of anthocyanins in NHB and RE blueberry [34, 69]. However, treatments with ABA at concentrations of 1000 mg l^−1^ produced leaf phytotoxicity in RE blueberries [34].

It appears that different fruit-ripening traits may require the use of multiple types of PGRs to ensure a positive effect on each trait. Thus, to date, there is not a single formulation that can accelerate, improve, or synchronize blueberry ripening. Additionally, the use of PGRs remains a subject of public concern due to real or perceived negative effects to human health and environmental preservation [82]. Managing ripening manipulation on a commercial scale through PGRs could be particularly challenging due to such complexities.

A high degree of ripening uniformity is crucial to improve hand or machine harvest efficiency. Luby and Finn [30] identified a positive relationship between ripening duration and crop load in a population of clones and hybrids of *V. corymbosum* and *V. angustifolium*. Also, Maust *et al*. [83] suggested that reducing the number of flower buds during the dormancy period can expedite ripening while improving fruit size and quality because flowers compete with vegetative buds for reserves after dormancy release [84]. As carbohydrate pools deplete, a disparity in budbreak occurs, leading to vegetative and floral organs developing at different times. Thus, crop load manipulation strategies to concentrate blueberry ripening should be applied before budbreak while plants are dormant. This reduces stress on the plants, as their growth is minimal or entirely absent during dormancy [85]. Other factors, such as late spring freezes, should be considered when implementing crop load manipulation strategies, as a severe freeze could damage the entire crop if fruiting is highly concentrated. Currently, effective and scalable methods for crop load manipulation in blueberries are unavailable.

While concentrated harvest has been previously identified as a breeding target for mechanically harvested blueberry [86], little progress has been made on the matter to date. The development of blueberry varieties with improved ripening has been mostly focused on breeding genotypes that avoid frost conditions that can damage flower buds [23]. Miyashita *et al*. [87] observed that parthenocarpic hybrids exhibited more uniform ripening compared to those producing seeded fruit. They concluded that parthenocarpic blueberries could be advantageous for cluster harvesting, enhancing harvestability and shelf life quality. However, Cullen *et al*. [88] found that the impact of parthenocarpy on ripening varies across different years, suggesting that further research is warranted in this area.

## Summary and future perspectives

The reviewed literature highlights persistent uncertainty regarding a crop-wide ripening classification of blueberries. Ripening traits in blueberries develop progressively—some traits enhance fruit value (e.g. sugar accumulation) while others reduce it (e.g. fruit softening). There seems to be great phenotypic diversity within the crop. Ethylene is produced throughout fruit development (Fig. 2). Initial fruit skin color changes appear to be influenced by ethylene. However, other ripening-associated traits such as soluble carbohydrates and organic acids accumulation predominantly occur when ethylene levels decrease. Future research should address the impact of endogenous and exogenous ethylene levels on individual fruit ripening traits (e.g. color, sugar accumulation, volatiles profile, etc.). Similar to melons, blueberries might exhibit distinct ethylene-dependent and ethylene-independent responses across different traits during ripening, highlighting the need for a more detailed and trait-specific approach to their study to develop targeted strategies to enhance traits of interest. While PGR applications and crop load strategies have been previously used for improving blueberry ripening [83, 89], further research is still needed before these strategies can be commercially implemented.

Emerging technologies provide new avenues to investigate the physiological and molecular mechanisms underlying blueberry ripening. High-throughput phenotyping using machine vision and deep learning can enable precise, nondestructive measurement of ripening traits across several genotypes. The ability to handle many samples and genotypes will be critical for genomic studies looking to investigate the genetic architecture of ripening traits. Although genetic and genomic profiles of ripening have been developed, metabolomic data remain limited. Integrating multi-omics approaches will be key to uncovering the genetic and biochemical basis of ripening. Collectively, these tools can improve our understanding of genotype- and trait-specific responses, informing the development of breeding ideotypes and agronomic strategies to enhance fruit quality, harvest efficiency, and shelf life.

## Supplementary Material

Web_Material_uhaf126

## Data Availability

The data used in this article are available in the online supplementary materials.
