# Supplementary material for: Blueberry ripening mechanism: a systematic review of physiological and molecular evidence
Source: Hortic Res. 2025 May 14;12(8):uhaf126. doi: 10.1093/hr/uhaf126 (PMC12261913; doi:10.1093/hr/uhaf126)
Supplement: Web_Material_uhaf126 [file web_material_uhaf126.zip › Supplementary Materials.docx]

**Supplementary Materials**

**Supplementary table 1.** Extraction methods of CO2 and C2H4 across various ripening studies using different blueberry types and species.

| **Year** | **Genotype** | **Species** | **Blueberry Type** | **Peak CO_2_ rate (mg kg^-1^ h^-1^)** | **Peak C_2_H_4_ rate (mg kg^-1^ h^-1^)** | **Ripening classification** | **Method of CO_2_ or C_2_H_4_ detection** | **Reference** |
| --- | --- | --- | --- | --- | --- | --- | --- | --- |
| 1929 | Rubel | *V. corymbosum* L. | NHB | NA | NA | Undefined | CO_2_ measured on green, CT and blue fruits over a period of 1 month | [52] |
| 1967 | NA | *V. angustifolium* Ait. | LB | NA | NA | NC | Oxygen consumption of CT and blue fruit enclosed in flasks was measured manometrically to estimate respiration per 1-h period with fruit samples kept at 25 °C. Maximum O^2^ consumption when fruits were blue. | [50] |
| 1967 | NA | *V. angustifolium* Ait. | LB | At 14 DAH: 37.9 At 21 DAH: 26.9 At 28 DAH: 29.2 | NA | CL | Respiration rate measured at the post-climacteric stage (14, 21 and 28 DAH) of blue fruits (*n*= 200 g) as CO_2_ evolution at 24-h intervals for a 96-h period using an infrared gas analyzer (Lira model 200, MSA®, Pittsburg, PA, USA) with fruit samples kept at 21 °C. | [53] |
| 1969 | 6 clones | *V. angustifolium* Ait. | LB | 15.5 °C= 26.4 21.0 °C= 38.8 25.5 °C= 58.4 | NA | CL | Respiration rate was measured in fruit samples (n = 200 g) at various maturity stages based on color (green, CT, and blue). CO₂ evolution was assessed at different temperatures (15.5, 21, and 25.5 °C) over a 96-hour period, with measurements taken at 24-hour intervals using an infrared gas analyzer (Lira model 200, MSA®, Pittsburg, PA, USA). | [3] |
| 1969 | Bluecrop | *V. corymbosum* L. | NHB | 38.8 | NA | CL | Respiration rate measured on fruit samples (*n*= 200 g) at different stages of maturity based on color (Green, CT and blue). CO_2_ evolution at 24-h intervals for a 96-h period using an infrared gas analyzer (Lira model 200, MSA®, Pittsburg, PA, USA) with fruit samples kept at 21 °C. | [3] |
| 1969 | Jersey | *V. corymbosum* L. | NHB | 70.0 | NA | CL | Respiration rate measured on fruit samples (*n*= 200 g) at different stages of maturity based on color (Green, CT and blue). CO_2_ evolution at 24-h intervals for a 96-h period using an infrared gas analyzer (Lira model 200, MSA®, Pittsburg, PA, USA) with fruit samples kept at 21 °C. | [3] |
| 1969 | Maine 5009 | *V. corymbosum* L. | NHB | 66.0 | NA | CL | Respiration rate measured on fruit samples (*n*= 200 g) at different stages of maturity based on color (Green, CT and blue). CO_2_ evolution at 24-h intervals for a 96-h period using an infrared gas analyzer (Lira model 200, MSA®, Pittsburg, PA, USA) with fruit samples kept at 21 °C. | [3] |
| 1969 | Rubel | *V. corymbosum* L. | NHB | 76.4 | NA | CL | Respiration rate measured on fruit samples (*n*= 200 g) at different stages of maturity based on color (Green, CT and blue). CO_2_ evolution at 24-h intervals for a 96-h period using an infrared gas analyzer (Lira model 200, MSA®, Pittsburg, PA, USA) with fruit samples kept at 21 °C. | [3] |
| 1972 | G-90 | *V. corymbosum* L. | NHB | 72.0 | 0.00005 | NC | CO_2_ and C_2_H_4_ evolution was measured on green, CT, and blue fruits (*n*= 20) enclosed in flasks at 0.5-h intervals. A gas chromatograph (model 609, F&M Scientific, Avondale, PA, USA) was used for C_2_H_4_ detection, whereas for CO_2_, a gas chromatogram (GCS, Beckman Coulter Inc., Brea, CA, USA) was used. | [48] |
| 1974 | NA | *V. angustifolium* Ait. | LB | NA | NA | NA | NA | [90] |
| 1976 | Bluecrop | *V. corymbosum* L. | NHB | 138.9 | 0.00046 | CL | CO₂ and C₂H₄ evolution were measured in green, CT, and blue fruits enclosed in flasks. CO₂ evolution was monitored in fruits (*n*= 50) using an infrared analyzer (model IR315, Beckman Coulter Inc., Brea, CA, USA) at 0.3-hour intervals over a 2-3 hour period. For C₂H₄ measurement, a gas chromatograph (model 1800, model 1800, Varian Inc., Walnut Creek, CA, USA) was used. Fruits (*n*= 3) were maintained at 23 ± 2 °C, and gas samples were collected 30 minutes after placement in the analysis tubes. | [36] |
| 1976 | Lateblue | *V. corymbosum* L. | NHB | 131.0 | 0.00050 | CL | CO₂ and C₂H₄ evolution were measured in green, CT, and blue fruits enclosed in flasks. CO₂ evolution was monitored in fruits (*n*= 50) using an infrared analyzer (model IR315, Beckman Coulter Inc., Brea, CA, USA) at 0.3-hour intervals over a 2-3 hour period. For C₂H₄ measurement, a gas chromatograph (model 1800, Varian Inc., Walnut Creek, CA, USA) was used. Fruits (*n*= 3) were maintained at 23 ± 2 °C, and gas samples were collected 30 minutes after placement in the analysis tubes. | [36] |
| 1978 | Coville | *V. corymbosum* L. | NHB | 86.0 | NA | NC | Respiration rate measured on harvested green-CT fruits (*n*= 200) as CO^2^ evolution at 5-h intervals for a 30-h period using an infrared analyzer (model 15A, Beckman Coulter Inc., Brea, CA, USA) with fruit samples kept at a constant temperature of 22 ± 2 °C during measurement. | [91] |
| 1978 | Earliblue | *V. corymbosum* L. | NHB | 112.2 | NA | NC | Respiration rate measured on harvested green-CT fruits (*n*= 200) as CO^2^ evolution at 5-h intervals for a 30-h period using an infrared analyzer (model 15A, Beckman Coulter Inc., Brea, CA, USA) with fruit samples kept at a constant temperature of 22 ± 2 °C during measurement. | [91] |
| 1982 | Delite | *V. virgatum* Ait; syn. *V. ashei* Reade | RE | NA | 0.18000 | CL | C_2_H_4_ evolution measured on green, CT, and blue fruits at 24-h intervals for an 8-day period using a gas chromatograph (model 200, Varian Inc., Walnut Creek, CA, USA) with fruit samples kept at a temperature of 18 °C. | [43] |
| 1986 | Jersey | *V. corymbosum* L. | NHB | 269.4 | 3.05882 | CL | CO₂ and C₂H₄ levels were measured in green, CT, and blue fruits (*n* = 10 g) enclosed in a desiccator. The fruits were incubated at a constant temperature of 23 ± 1 °C for 3-4 hours. CO₂ was measured using an infrared analyzer (model URA-3B, Shimadzu Co., Kyoto, Japan), while C₂H₄ was determined using a gas chromatograph (model GC-7A, Shimadzu Co., Kyoto, Japan). | [44] |
| 1986 | Tifblue | *V. virgatum* Ait; syn. *V. ashei* Reade | RE | 151.8 | 3.14286 | CL | CO₂ and C₂H₄ levels were measured in green, CT, and blue fruits (*n* = 10 g) enclosed in a desiccator. The fruits were incubated at a constant temperature of 23 ± 1 °C for 3-4 hours. CO₂ was measured using an infrared analyzer (model URA-3B, Shimadzu Co., Kyoto, Japan), while C₂H₄ was determined using a gas chromatograph (model GC-7A, Shimadzu Co., Kyoto, Japan). | [44] |
| 1986 | Weymouth | *V. corymbosum* L. | NHB | 132.6 | 2.03448 | CL | CO₂ and C₂H₄ levels were measured in green, CT, and blue fruits (*n* = 10 g) enclosed in a desiccator. The fruits were incubated at a constant temperature of 23 ± 1 °C for 3-4 hours. CO₂ was measured using an infrared analyzer (model URA-3B, Shimadzu Co., Kyoto, Japan), while C₂H₄ was determined using a gas chromatograph (model GC-7A, Shimadzu Co., Kyoto, Japan). | [44] |
| 1986 | Woodard | *V. virgatum* Ait; syn. *V. ashei* Reade | RE | 212.0 | 7.00000 | CL | CO₂ and C₂H₄ levels were measured in green, CT, and blue fruits (*n* = 10 g) enclosed in a desiccator. The fruits were incubated at a constant temperature of 23 ± 1 °C for 3-4 hours. CO₂ was measured using an infrared analyzer (model URA-3B, Shimadzu Co., Kyoto, Japan), while C₂H₄ was determined using a gas chromatograph (model GC-7A, Shimadzu Co., Kyoto, Japan). | [44] |
| 1997 | Berkeley | *V. corymbosum* L. | NHB | 315.1 | 0.00173 | Undefined | Fruits (*n*= 2-10) of different maturity stages (green, CT and blue) were placed in a sealed bottle for six hours at a constant temperature 20-25 °C. Then, C_2_H_4_ was analyzed using a flame ionization detector-gas chromatographer (model DC-14A, Shimadzu Co., Kyoto, Japan). Similarly, these fruits were sampled for CO_2_ determination using a thermal conductivity detector-gas chromatography. | [45] |
| 1997 | Collins | *V. corymbosum* L. | NHB | 381.3 | 0.00309 | Undefined | Fruits (*n*= 2-10) of different maturity stages (green, CT and blue) were placed in a sealed bottle for six hours at a constant temperature 20-25 °C. Then, C_2_H_4_ was analyzed using a flame ionization detector-gas chromatographer (model DC-14A, Shimadzu Co., Kyoto, Japan). Similarly, these fruits were sampled for CO_2_ determination using a thermal conductivity detector-gas chromatography. | [45] |
| 1997 | Dixi | *V. corymbosum* L. | NHB | 277.3 | 0.00082 | Undefined | Fruits (*n*= 2-10) of different maturity stages (green, CT and blue) were placed in a sealed bottle for six hours at a constant temperature 20-25 °C. Then, C_2_H_4_ was analyzed using a flame ionization detector-gas chromatographer (model DC-14A, Shimadzu Co., Kyoto, Japan). Similarly, these fruits were sampled for CO_2_ determination using a thermal conductivity detector-gas chromatography. | [45] |
| 2012 | Star | *V. corymbosum* L. interspecific hybrids | SHB | NA | NA | NC | NA | [89] |
| 2012 | Windsor | *V. corymbosum* L. interspecific hybrids | SHB | NA | NA | NC | NA | [89] |
| 2012 | Rubel | *V. corymbosum* L. | NHB | NA | NA | NC | NA | [4] |
| 2014 | Star | *V. corymbosum* L. interspecific hybrids | SHB | NA | NA | CL | NA | [77] |
| 2014 | Sweetcrisp | *V. corymbosum* L. interspecific hybrids | SHB | NA | NA | CL | NA | [77] |
| 2018 | Jersey | *V. corymbosum* L. | NHB | NA | NA | NC | NA | [92] |
| 2018 | Powderblue | *V. virgatum* Ait; syn. *V. ashei* Reade | RE | NA | NA | Undefined | NA | [34] |
| 2018 | Premier | *V. virgatum* Ait; syn. *V. ashei* Reade | RE | NA | NA | Undefined | NA | [34] |
| 2019 | Bluecrop | *V. corymbosum* L. | NHB | NA | NA | NC | NA | [68] |
| 2021 | Jersey | *V. corymbosum* L. | NHB | NA | 0.00419 | CL | Fruits (*n*= 3-4) were placed in a sealed bottle for six hours at constant room temperature (25 °C). Ethylene was measured at 72-h intervals for a 16-day period using a gas chromatographer (model GC-14A, Shimadzu Co., Kyoto, Japan). | [46] |
| 2022 | Atlantic | *V. corymbosum* L. | NHB | NA | 0.00662 | Undefined | Fruits (*n*= 3) were placed in a sealed glass vial for four hours at constant temperature (21 °C). Ethylene was measured on fruits with different maturity stages (green, CT and blue) using a proton transfer reaction - mass spectrometer (model 8000, model 8000, Ionicon Analytik GmbH, Innsbruck, Austria) with the O_2_^+^ mode. | [47] |
| 2022 | Berkeley | *V. corymbosum* L. | NHB | NA | 0.00106 | Undefined | Fruits (*n*= 3) were placed in a sealed glass vial for four hours at constant temperature (21 °C). Ethylene was measured on fruits with different maturity stages (green, CT and blue) using a proton transfer reaction - mass spectrometer (model 8000, model 8000, Ionicon Analytik GmbH, Innsbruck, Austria) with the O_2_^+^ mode. | [47] |
| 2022 | Biloxi | *V. corymbosum* L. interspecific hybrids | SHB | NA | 0.00117 | Undefined | Fruits (*n*= 3) were placed in a sealed glass vial for four hours at constant temperature (21 °C). Ethylene was measured on fruits with different maturity stages (green, CT and blue) using a proton transfer reaction - mass spectrometer (model 8000, model 8000, Ionicon Analytik GmbH, Innsbruck, Austria) with the O_2_^+^ mode. | [47] |
| 2022 | Bluechip | *V. corymbosum* L. | NHB | NA | 0.00087 | Undefined | Fruits (*n*= 3) were placed in a sealed glass vial for four hours at constant temperature (21 °C). Ethylene was measured on fruits with different maturity stages (green, CT and blue) using a proton transfer reaction - mass spectrometer (model 8000, Ionicon Analytik GmbH, Innsbruck, Austria) with the O_2_^+^ mode. | [47] |
| 2022 | Bluegold | *V. corymbosum* L. | NHB | NA | 0.00121 | Undefined | Fruits (*n*= 3) were placed in a sealed glass vial for four hours at constant temperature (21 °C). Ethylene was measured on fruits with different maturity stages (green, CT and blue) using a proton transfer reaction - mass spectrometer (model 8000, model 8000, Ionicon Analytik GmbH, Innsbruck, Austria) with the O_2_^+^ mode. | [47] |
| 2022 | Brigitta | *V. corymbosum* L. | NHB | NA | 0.00098 | Undefined | Fruits (*n*= 3) were placed in a sealed glass vial for four hours at constant temperature (21 °C). Ethylene was measured on fruits with different maturity stages (green, CT and blue) using a proton transfer reaction - mass spectrometer (model 8000, model 8000, Ionicon Analytik GmbH, Innsbruck, Austria) with the O_2_^+^ mode. | [47] |
| 2022 | Chandler | *V. corymbosum* L. | NHB | NA | 0.00147 | Undefined | Fruits (*n*= 3) were placed in a sealed glass vial for four hours at constant temperature (21 °C). Ethylene was measured on fruits with different maturity stages (green, CT and blue) using a proton transfer reaction - mass spectrometer (model 8000, model 8000, Ionicon Analytik GmbH, Innsbruck, Austria) with the O_2_^+^ mode. | [47] |
| 2022 | Emerald | *V. corymbosum* L. interspecific hybrids | SHB | NA | 0.00151 | Undefined | Fruits (*n*= 3) were placed in a sealed glass vial for four hours at constant temperature (21 °C). Ethylene was measured on fruits with different maturity stages (green, CT and blue) using a proton transfer reaction - mass spectrometer (model 8000, model 8000, Ionicon Analytik GmbH, Innsbruck, Austria) with the O_2_^+^ mode. | [47] |
| 2022 | Jersey | *V. corymbosum* L. | NHB | NA | 0.00491 | Undefined | Fruits (*n*= 3) were placed in a sealed glass vial for four hours at constant temperature (21 °C). Ethylene was measured on fruits with different maturity stages (green, CT and blue) using a proton transfer reaction - mass spectrometer (model 8000, model 8000, Ionicon Analytik GmbH, Innsbruck, Austria) with the O_2_^+^ mode. | [47] |
| 2022 | Jubilee | *V. corymbosum* L. interspecific hybrids | SHB | NA | 0.00072 | Undefined | Fruits (*n*= 3) were placed in a sealed glass vial for four hours at constant temperature (21 °C). Ethylene was measured on fruits with different maturity stages (green, CT and blue) using a proton transfer reaction - mass spectrometer (model 8000, model 8000, Ionicon Analytik GmbH, Innsbruck, Austria) with the O_2_^+^ mode. | [47] |
| 2022 | Legacy | *V. corymbosum* L. interspecific hybrids | SHB | NA | 0.00132 | Undefined | Fruits (*n*= 3) were placed in a sealed glass vial for four hours at constant temperature (21 °C). Ethylene was measured on fruits with different maturity stages (green, CT and blue) using a proton transfer reaction - mass spectrometer (model 8000, Ionicon Analytik GmbH, Innsbruck, Austria) with the O_2_^+^ mode. | [47] |
| 2022 | Misty | *V. corymbosum* L. interspecific hybrids | SHB | NA | 0.00132 | Undefined | Fruits (*n*= 3) were placed in a sealed glass vial for four hours at constant temperature (21 °C). Ethylene was measured on fruits with different maturity stages (green, CT and blue) using a proton transfer reaction - mass spectrometer (model 8000, model 8000, Ionicon Analytik GmbH, Innsbruck, Austria) with the O_2_^+^ mode. | [47] |
| 2022 | Brigitta | *V. corymbosum* L. | NHB | 36.4 | 0.00068 | Undefined | Fruits (*n*= 3) of different maturity stages (green, CT, and blue) were placed in sealed glass containers for 2 hours at 18 °C. For CO_2_ rate a gas analyzer (Quantek 902P, Quantek Instruments Inc., MA, USA) was used. For C₂H₄ evolution, 1 ml of the gas in each glass container was analyzed using a gas chromatograph (GC-17A, Shimadzu, Kyoto, Japan) equipped with a flame ionization detector. Ijector, oven and detector temperatures were set at 75 °C, 100 °C and 170 °C, respectively. | [93] |
| 2022 | Duke | *V. corymbosum* L. | NHB | 69.8 | 0.00230 | Undefined | Fruits (*n*= 3) of different maturity stages (green, CT, and blue) were placed in sealed glass containers for 2 hours at 18 °C. For CO_2_ rate a gas analyzer (Quantek 902P, Quantek Instruments Inc., MA, USA) was used. For C₂H₄ evolution, 1 ml of the gas in each glass container was analyzed using a gas chromatograph (GC-17A, Shimadzu, Kyoto, Japan) equipped with a flame ionization detector. Ijector, oven and detector temperatures were set at 75 °C, 100 °C and 170 °C, respectively. | [93] |
| 2022 | Alapaha | *V. virgatum* Ait; syn. *V. ashei* Reade | RE | 115.1 | NA | Atypical CL | Fruits were harvested at different maturity stages (green, CT, and blue) and stored overnight at 4 °C with approximately 90% relative humidity before CO₂ and C₂H₄ measurements. For CO₂ analysis, about 10 g of fruit were placed in a glass jar with a septum and incubated for 1 hour at 23 °C, followed by analysis using a gas analyzer (model 902P, Quantek Instruments Inc., MA, USA). For C₂H₄ evolution, 25 g of fruit were sealed in an airtight glass jar fitted with a rubber septum and incubated for 4 hours. Gas samples were then taken and analyzed with a gas chromatograph (model GC-17A, Shimadzu Co., Kyoto, Japan) equipped with a flame ionization detector. | [9] |
| 2022 | Brightwell | *V. virgatum* Ait; syn. *V. ashei* Reade | RE | 134.7 | 0.00135 | Atypical CL | Fruits were harvested at different maturity stages (green, CT, and blue) and stored overnight at 4 °C with approximately 90% relative humidity before CO₂ and C₂H₄ measurements. For CO₂ analysis, about 10 g of fruit were placed in a glass jar with a septum and incubated for 1 hour at 23 °C, followed by analysis using a gas analyzer (model 902P, Quantek Instruments Inc., MA, USA). For C₂H₄ evolution, 25 g of fruit were sealed in an airtight glass jar fitted with a rubber septum and incubated for 4 hours. Gas samples were then taken and analyzed with a gas chromatograph (model GC-17A, Shimadzu Co., Kyoto, Japan) equipped with a flame ionization detector. | [9] |
| 2022 | Emerald | *V. corymbosum* L. interspecific hybrids | SHB | 162.8 | 0.00062 | Atypical CL | Fruits were harvested at different maturity stages (green, CT, and blue) and stored overnight at 4 °C with approximately 90% relative humidity before CO₂ and C₂H₄ measurements. For CO₂ analysis, about 10 g of fruit were placed in a glass jar with a septum and incubated for 1 hour at 23 °C, followed by analysis using a gas analyzer (model 902P, Quantek Instruments Inc., MA, USA). For C₂H₄ evolution, 25 g of fruit were sealed in an airtight glass jar fitted with a rubber septum and incubated for 4 hours. Gas samples were then taken and analyzed with a gas chromatograph (model GC-17A, Shimadzu Co., Kyoto, Japan) equipped with a flame ionization detector. | [9] |
| 2022 | Krewer | *V. virgatum* Ait; syn. *V. ashei* Reade | RE | 120.7 | NA | Atypical CL | Fruits were harvested at different maturity stages (green, CT, and blue) and stored overnight at 4 °C with approximately 90% relative humidity before CO₂ and C₂H₄ measurements. For CO₂ analysis, about 10 g of fruit were placed in a glass jar with a septum and incubated for 1 hour at 23 °C, followed by analysis using a gas analyzer (model 902P, Quantek Instruments Inc., MA, USA). For C₂H₄ evolution, 25 g of fruit were sealed in an airtight glass jar fitted with a rubber septum and incubated for 4 hours. Gas samples were then taken and analyzed with a gas chromatograph (model GC-17A, Shimadzu Co., Kyoto, Japan) equipped with a flame ionization detector. | [9] |
| 2022 | Miss Alice Mae | *V. corymbosum* L. interspecific hybrids | SHB | 174.0 | NA | Atypical CL | Fruits were harvested at different maturity stages (green, CT, and blue) and stored overnight at 4 °C with approximately 90% relative humidity before CO₂ and C₂H₄ measurements. For CO₂ analysis, about 10 g of fruit were placed in a glass jar with a septum and incubated for 1 hour at 23 °C, followed by analysis using a gas analyzer (model 902P, Quantek Instruments Inc., MA, USA). For C₂H₄ evolution, 25 g of fruit were sealed in an airtight glass jar fitted with a rubber septum and incubated for 4 hours. Gas samples were then taken and analyzed with a gas chromatograph (model GC-17A, Shimadzu Co., Kyoto, Japan) equipped with a flame ionization detector. | [9] |
| 2022 | Miss Jackie | *V. corymbosum* L. interspecific hybrids | SHB | 134.7 | 0.00163 | Atypical CL | Fruits were harvested at different maturity stages (green, CT, and blue) and stored overnight at 4 °C with approximately 90% relative humidity before CO₂ and C₂H₄ measurements. For CO₂ analysis, about 10 g of fruit were placed in a glass jar with a septum and incubated for 1 hour at 23 °C, followed by analysis using a gas analyzer (model 902P, Quantek Instruments Inc., MA, USA). For C₂H₄ evolution, 25 g of fruit were sealed in an airtight glass jar fitted with a rubber septum and incubated for 4 hours. Gas samples were then taken and analyzed with a gas chromatograph (model GC-17A, Shimadzu Co., Kyoto, Japan) equipped with a flame ionization detector. | [9] |
| 2022 | Miss Lily | *V. corymbosum* L. interspecific hybrids | SHB | 188.0 | 0.00153 | Atypical CL | Fruits were harvested at different maturity stages (green, CT, and blue) and stored overnight at 4 °C with approximately 90% relative humidity before CO₂ and C₂H₄ measurements. For CO₂ analysis, about 10 g of fruit were placed in a glass jar with a septum and incubated for 1 hour at 23 °C, followed by analysis using a gas analyzer (model 902P, Quantek Instruments Inc., MA, USA). For C₂H₄ evolution, 25 g of fruit were sealed in an airtight glass jar fitted with a rubber septum and incubated for 4 hours. Gas samples were then taken and analyzed with a gas chromatograph (model GC-17A, Shimadzu Co., Kyoto, Japan) equipped with a flame ionization detector. | [9] |
| 2022 | Powderblue | *V. virgatum* Ait; syn. *V. ashei* Reade | RE | 199.2 | 0.00059 | Atypical CL | Fruits were harvested at different maturity stages (green, CT, and blue) and stored overnight at 4 °C with approximately 90% relative humidity before CO₂ and C₂H₄ measurements. For CO₂ analysis, about 10 g of fruit were placed in a glass jar with a septum and incubated for 1 hour at 23 °C, followed by analysis using a gas analyzer (model 902P, Quantek Instruments Inc., MA, USA). For C₂H₄ evolution, 25 g of fruit were sealed in an airtight glass jar fitted with a rubber septum and incubated for 4 hours. Gas samples were then taken and analyzed with a gas chromatograph (model GC-17A, Shimadzu Co., Kyoto, Japan) equipped with a flame ionization detector. | [9] |
| 2022 | Premier | *V. virgatum* Ait; syn. *V. ashei* Reade | RE | 174.0 | 0.00187 | Atypical CL | Fruits were harvested at different maturity stages (green, CT, and blue) and stored overnight at 4 °C with approximately 90% relative humidity before CO₂ and C₂H₄ measurements. For CO₂ analysis, about 10 g of fruit were placed in a glass jar with a septum and incubated for 1 hour at 23 °C, followed by analysis using a gas analyzer (model 902P, Quantek Instruments Inc., MA, USA). For C₂H₄ evolution, 25 g of fruit were sealed in an airtight glass jar fitted with a rubber septum and incubated for 4 hours. Gas samples were then taken and analyzed with a gas chromatograph (model GC-17A, Shimadzu Co., Kyoto, Japan) equipped with a flame ionization detector. | [9] |
| 2022 | Rebel | *V. corymbosum* L. interspecific hybrids | SHB | 179.6 | 0.00173 | Atypical CL | Fruits were harvested at different maturity stages (green, CT, and blue) and stored overnight at 4 °C with approximately 90% relative humidity before CO₂ and C₂H₄ measurements. For CO₂ analysis, about 10 g of fruit were placed in a glass jar with a septum and incubated for 1 hour at 23 °C, followed by analysis using a gas analyzer (model 902P, Quantek Instruments Inc., MA, USA). For C₂H₄ evolution, 25 g of fruit were sealed in an airtight glass jar fitted with a rubber septum and incubated for 4 hours. Gas samples were then taken and analyzed with a gas chromatograph (model GC-17A, Shimadzu Co., Kyoto, Japan) equipped with a flame ionization detector. | [9] |
| 2022 | Suzieblue | *V. corymbosum* L. interspecific hybrids | SHB | 218.9 | 0.00093 | Atypical CL | Fruits were harvested at different maturity stages (green, CT, and blue) and stored overnight at 4 °C with approximately 90% relative humidity before CO₂ and C₂H₄ measurements. For CO₂ analysis, about 10 g of fruit were placed in a glass jar with a septum and incubated for 1 hour at 23 °C, followed by analysis using a gas analyzer (model 902P, Quantek Instruments Inc., MA, USA). For C₂H₄ evolution, 25 g of fruit were sealed in an airtight glass jar fitted with a rubber septum and incubated for 4 hours. Gas samples were then taken and analyzed with a gas chromatograph (model GC-17A, Shimadzu Co., Kyoto, Japan) equipped with a flame ionization detector. | [9] |
| 2022 | Titan | *V. virgatum* Ait; syn. *V. ashei* Reade | RE | 117.9 | 0.00229 | Atypical CL | Fruits were harvested at different maturity stages (green, CT, and blue) and stored overnight at 4 °C with approximately 90% relative humidity before CO₂ and C₂H₄ measurements. For CO₂ analysis, about 10 g of fruit were placed in a glass jar with a septum and incubated for 1 hour at 23 °C, followed by analysis using a gas analyzer (model 902P, Quantek Instruments Inc., MA, USA). For C₂H₄ evolution, 25 g of fruit were sealed in an airtight glass jar fitted with a rubber septum and incubated for 4 hours. Gas samples were then taken and analyzed with a gas chromatograph (model GC-17A, Shimadzu Co., Kyoto, Japan) equipped with a flame ionization detector. | [9] |
| 2024 | Powderblue | *V. virgatum* Ait; syn. *V. ashei* Reade | RE | NA | 0.00040 | Atypical CL | Fruits were harvested at different maturity stages (green, CT, and blue) and stored overnight at 4 °C with approximately 90% relative humidity before CO₂ and C₂H₄ measurements. For CO₂ analysis, about 10 g of fruit were placed in a glass jar with a septum and incubated for 1 hour at 23 °C, followed by analysis using a gas analyzer (model 902P, Quantek Instruments Inc., MA, USA). For C₂H₄ evolution, 25 g of fruit were sealed in an airtight glass jar fitted with a rubber septum and incubated for 4 hours. Gas samples were then taken and analyzed with a gas chromatograph (model GC-17A, Shimadzu Co., Kyoto, Japan) equipped with a flame ionization detector. | [9] |

NA = not available**,** RE = Rabbiteye Blueberry, LB = Lowbush Blueberry, SHB = Southern Highbush Blueberry, NHB = Northern Highbush Blueberry**,** CT = color transition**,** DAH = days after harvest**,** CL = climacteric**, and** NC = non-climacteric

Supplementary table 2. Function of some of the main genes involved in *Vaccinium* spp. fruit development.

| **Gene** | **Abbreviation** | **Action/Involved in** | **Vaccinium species** | **Reference** |
| --- | --- | --- | --- | --- |
| Abscisic aldehyde oxydase 3 | *VcAAO3* | ABA biosynthesis | *V. corymbosum* | [68] |
| ABA-responsive element binding factor | *VcABF* | ABA signal transduction | *V. corymbosum* | [68] |
| ATP-citrate lyase | *VcACLY* | Organic acid regulation | *V. corymbosum* | [70] |
| 1-aminocyclopropane-1- carboxylic acid oxidase 1 | *VaACO1* | Ethylene production pathway | *V. ashei* | [9] |
| 1-aminocyclopropane-1- carboxylic acid oxidase 2 | *VaACO2* | Ethylene production pathway | *V. ashei* | [9] |
| 1-aminocyclopropane-1- carboxylic acid oxidase 6 | *VcACO6* | Ethylene production pathway | *V. corymbosum* | [65] |
| 1-aminocyclopropane-1- carboxylic acid synthase 1 | *VaACS1*  *VcACS1* | Ethylene production pathway | *V. ashei*  *V. corymbosum* | [9,65] |
| Alcohol dehydrogenase | *VcADH* | Organic acid regulation | *V. corymbosum* | [70] |
| Anthocyanidin reductase | *VcANR* | Proanthocyanidin biosynthesis | *V. corymbosum* | [4,68] |
| Anthocyanidin synthase | *VcANS* | Anthocyanin biosynthesis | *V. corymbosum* | [4,68] |
| Auxin regulated gene involved organ size 2 | *VaARGOS2* | Ethylene signaling | *V. ashei* | [9] |
| ß-carotene 3-hydroxylase | *VcBCH* | ABA biosynthesis | *V. corymbosum* | [68] |
| Basic helix-loop-helix | *VcbHLH* | Anthocyanin biosynthesis | *V. corymbosum* | [68,94,95] |
| Carotenoid cleavage dioxygenase 1 | *VcCCD1* | Carotenoid breakdown | *V. corymbosum* | [51] |
| Chalcone isomerase | *VcCHI* | Anthocyanin biosynthesis | *V. corymbosum*  *V. angustifolium*×*V. corymbosum* | [68,96] |
| Chalcone synthase | *VcCHS* | Flavonoid pathway | *V. corymbosum* | [4,68,97] |
| Chalcone synthase 1 | *VcCHS1*  *VaCHS1* | Flavonoid pathway | *V. corymbosum*  *V. ashei* | [59] |
| Chalcone synthase 2 | *VcCHS2*  *VaCHS2* | Flavonoid pathway | *V. corymbosum*  *V. ashei* | [59] |
| β-carotene hydroxylase/Lutein deficient 5 | *VcCHYB/LUT5* | Xanthophyll biosynthesis | *V. corymbosum* | [51] |
| ε-hydroxylase/carotene ε-monooxygenase | *VcCHYE/LUT1* | Xanthophyll biosynthesis | *V. corymbosum* | [51] |
| Cytochrome b5 | *VcCytob5* | Anthocyanin biosynthesis | *V. corymbosum* | [4] |
| Dihydroflavonol 4-reductase | *VcDFR* | Proanthocyanidin and anthocyanin production pathways | *V. corymbosum* | [4,68] |
| Divinyl reductase | *VcDVR* | Chlorophyll biosynthesis | *V. corymbosum* | [65] |
| Ethylene insensitive 3 | *VaEIN3* | Ethylene signaling | *V. ashei* | [9] |
| Ethylene receptor 1 | *VaETR1* | Ethylene signaling | *V. ashei* | [9] |
| Ethylene receptor 3/4 | *VaETR3/4* | Ethylene signaling | *V. ashei* | [9] |
| Flavonoid 3', 5' -hydroxylase | *VcF3’5’H* | Flavonoid pathway | *V. corymbosum* | [4,68] |
| Flavanone 3-hydroxylase | *VcF3H* | Flavonoid pathway | *V. corymbosum* | [68] |
| Flavonoid 3' - hydroxylase | *VcF3'H* | Flavonoid pathway | *V. corymbosum* | [4,68] |
| Flavanone-3β-hydroxylase | *VcFHT* | Flavonoid pathway | *V. corymbosum* | [4] |
| Glutamate decarboxylase | *VcGAD* | Organic acid regulation | *V. corymbosum* | [70] |
| Glutamate synthase | *VcGLT* | Organic acid regulation | *V. corymbosum* | [70] |
| Aux/IAA family gene | *VcIAA27* | Fruit enlargement |  | [58] |
| Invertase | *VcINV* | Sugar accumulation | *V. corymbosum* | [70] |
| Leucoanthocyanidin reductase | *VcLAR* | Proanthocyanidin biosynthesis | *V. corymbosum* | [4,68] |
| Malate dehydrogenase | *VcMDH* | Organic acid regulation | *V. corymbosum* | [70] |
| Subgroup-6 R2R3MYB protein homolog 102 | *VcMYB102* | Anthocyanin biosynthesis | *V. corymbosum* | [63] |
| Subgroup-6 R2R3MYB protein homolog 104 | *VcMYB104* | Anthocyanin biosynthesis | *V. corymbosum* | [63] |
| Subgroup-6 R2R3MYB protein homolog 115 | *VcMYB115* | Anthocyanin biosynthesis | *V. corymbosum* | [63] |
| Subgroup-6 R2R3MYB protein homolog 13 | *VcMYB13* | Anthocyanin biosynthesis | *V. corymbosum* | [63] |
| Subgroup-6 R2R3MYB protein homolog 192 | *VcMYB192* | Anthocyanin biosynthesis | *V. corymbosum* | [63] |
| Subgroup-6 R2R3MYB protein homolog 219 | *VcMYB219* | Anthocyanin biosynthesis | *V. corymbosum* | [63] |
| Subgroup-6 R2R3MYB protein homolog 336 | *VcMYB336* | Anthocyanin biosynthesis | *V. corymbosum* | [63] |
| Subgroup-6 R2R3MYB protein homolog 35 | *VcMYB35* | Anthocyanin biosynthesis | *V. corymbosum* | [63] |
| Subgroup-6 R2R3MYB protein homolog 369 | *VcMYB369* | Anthocyanin biosynthesis | *V. corymbosum* | [63] |
| Subgroup-6 R2R3MYB protein homolog 433 | *VcMYB433* | Anthocyanin biosynthesis | *V. corymbosum* | [63] |
| Subgroup-6 R2R3MYB protein homolog 49 | *VcMYB49* | Anthocyanin biosynthesis | *V. corymbosum* | [63] |
| Subgroup-6 R2R3MYB protein homolog 72 | *VcMYB72* | Anthocyanin biosynthesis | *V. corymbosum* | [63] |
| Subgroup-6 R2R3MYB protein homolog 89 | *VcMYB89* | Anthocyanin biosynthesis | *V. corymbosum* | [63] |
| Subgroup-6 R2R3MYB protein homolog 1 | *VcMYBPA1* | Proanthocyanidin biosynthesis | *V. corymbosum* | [4,63,69] |
| NADP (Nicotinamide adenine dinucleotide phosphate)-dependent malic enzyme | *VcNADP-ME* | Organic acid regulation | *V. corymbosum* | [70] |
| 9-cis-epoxycarotenoid dioxygenase | *VcNCED* | ABA biosynthesis | *V. corymbosum* | [68] |
| 9-cis-epoxycarotenoid dioxygenase 1 | *VcNCED1* | ABA biosynthesis | *V. corymbosum* | [4] |
| 9-cis-epoxycarotenoid dioxygenase 2 | *VcNCED2*  *VaNCED2* | ABA biosynthesis | *V. corymbosum*  *V. ashei* | [59] |
| Pyruvate decarboxylase | *VcPDC* | Organic acid regulation | *V. corymbosum* | [70] |
| Phytoene desaturase | *VcPDS* | Carotene biosynthesis | *V. corymbosum* | [51] |
| Pectinesterase1 | *VcPE1*  *VaPE1* | Cell wall metabolism (turnover and loosing) | *V. corymbosum*  *V. ashei* | [59] |
| Protein phosphatases type 2C | *VcPP2C* | ABA signal transduction | *V. corymbosum* | [68] |
| Phytoene synthase | *VcPSY* | Carotene biosynthesis | *V. corymbosum* | [51] |
| Pyrabactin resistance-like | *VcPYL* | ABA signal transduction | *V. corymbosum* | [68] |
| Reversion to ethylene sensitivity 1 | *VaRTE1* | Ethylene signaling | *V. ashei* | [9] |
| SQUAMOSA Promoter Binding Protein 10 | *VcSBP10* | Chlorophyll biosynthesis | *V. corymbosum* | [62] |
| SQUAMOSA Promoter Binding Protein 12a | *VcSBP12a* | Chlorophyll biosynthesis | *V. corymbosum* | [62] |
| SQUAMOSA Promoter Binding Protein 12b | *VcSBP12b* | Chlorophyll biosynthesis | *V. corymbosum* | [62] |
| SQUAMOSA Promoter Binding Protein 13a | *VcSBP13a* | Chlorophyll biosynthesis | *V. corymbosum* | [62] |
| SQUAMOSA Promoter Binding Protein 13b | *VcSBP13b* | Chlorophyll biosynthesis | *V. corymbosum* | [62] |
| SQUAMOSA Promoter Binding Protein 14a | *VcSBP14a* | Chlorophyll biosynthesis | *V. corymbosum* | [62] |
| SQUAMOSA Promoter Binding Protein 14aAS | *VcSBP14aAS* | Chlorophyll biosynthesis | *V. corymbosum* | [62] |
| SQUAMOSA Promoter Binding Protein 2 | *VcSBP2* | Chlorophyll biosynthesis | *V. corymbosum* | [62] |
| SQUAMOSA Promoter Binding Protein 3 | *VcSBP3* | Chlorophyll biosynthesis | *V. corymbosum* | [62] |
| SQUAMOSA Promoter Binding Protein 5 | *VcSBP5* | Chlorophyll biosynthesis | *V. corymbosum* | [62] |
| SQUAMOSA Promoter Binding Protein 6a | *VcSBP6a* | Chlorophyll biosynthesis | *V. corymbosum* | [62] |
| SQUAMOSA Promoter Binding Protein 6b | *VcSBP6b* | Chlorophyll biosynthesis | *V. corymbosum* | [62] |
| SQUAMOSA Promoter Binding Protein 7a | *VcSBP7a* | Chlorophyll biosynthesis | *V. corymbosum* | [62] |
| SQUAMOSA Promoter Binding Protein 8a | *VcSBP8a* | Chlorophyll biosynthesis | *V. corymbosum* | [62] |
| SQUAMOSA Promoter Binding Protein 8b | *VcSBP8b* | Chlorophyll biosynthesis | *V. corymbosum* | [62] |
| SQUAMOSA Promoter Binding Protein 9a | *VcSBP9a* | Chlorophyll biosynthesis | *V. corymbosum* | [62] |
| SQUAMOSA Promoter Binding Protein 9b | *VcSBP9b* | Chlorophyll biosynthesis | *V. corymbosum* | [62] |
| Sucrose non-fermenting-1-related protein kinase 2 | *VcSnRK2* | ABA signal transduction | *V. corymbosum* | [68] |
| SQUAMOSA promoter binding proteinlike | *VcSPL12* | Chlorophyll biosynthesis and breakdown | *V. corymbosum* | [65,98] |
| Sucrose-phosphatase | *VcSPP* | Sugar accumulation | *V. corymbosum* | [70] |
| S-adenosylmethionine synthase | *VaSAMS* | S-adenosylmethionine synthesis in the ethylene biosynthesis pathway | *V. ashei* | [64] |
| Sucrose-phosphate synthase | *VcSPS* | Sugar accumulation | *V. corymbosum* | [70] |
| SQUAMOSA-class MADS box | *VmTDR* | Anthocyanin biosynthesis | *V. corymbosum* | [68] |
| SQUAMOSA-class MADS box | *VmTDR4* | Anthocyanin biosynthesis | *V. myrtillus* | [99] |
| UDP-Glucose: Flavonoid-3-O-Glycosyltransferase | *VcUFGT* | Anthocyanin biosynthesis | *V. corymbosum* | [4,68] |
| Anthocyanidin 3-O-Glycosyltransferase 1 | *VaUFGT1* | Anthocyanin biosynthesis | *V. ashei* | [59] |
| Anthocyanidin 3-O-Glycosyltransferase 2 | *VaUFGT2* | Anthocyanin biosynthesis | *V. ashei* | [59,100] |
| Violxanthin de-epoxidase | *VcVDE* | Xanthophyll biosynthesis | *V. corymbosum* | [51] |
| Xyloglucan endotransglucosylase/hydrolase 1 | *VcXTH1*  *VaXTH1* | Cell wall metabolism (turnover and loosing) | *V. corymbosum*  *V. ashei* | [59] |
| Xyloglucan endotransglucosylase/hydrolase 2 | *VcXTH2*  *VaXTH2* | Cell wall metabolism (turnover and loosing) | *V. corymbosum*  *V. ashei* | [59] |
| Zeaxanthin epoxidase | *VcZEP* | Xanthophyll biosynthesis | *V. corymbosum* | [51] |
| ζ-carotene isomerase | *VcZ-ISO* | Carotene biosynthesis | *V. corymbosum* | [51] |
| β-Galactosidase 1 | *VcbGAL1*  *VabGAL1* | Cell wall metabolism (turnover and loosing) | *V. corymbosum*  *V. ashei* | [59] |
| 1,4-β-Mannosidase 1 | *VcbMAN1*  *VabMAN1* | Cell wall metabolism (turnover and loosing) | *V. corymbosum*  *V. ashei* | [59] |

*Vc*: *Vaccinium corymbosum Va*: *Vaccinium ashei Vm*: *Vaccinium myrtillus*


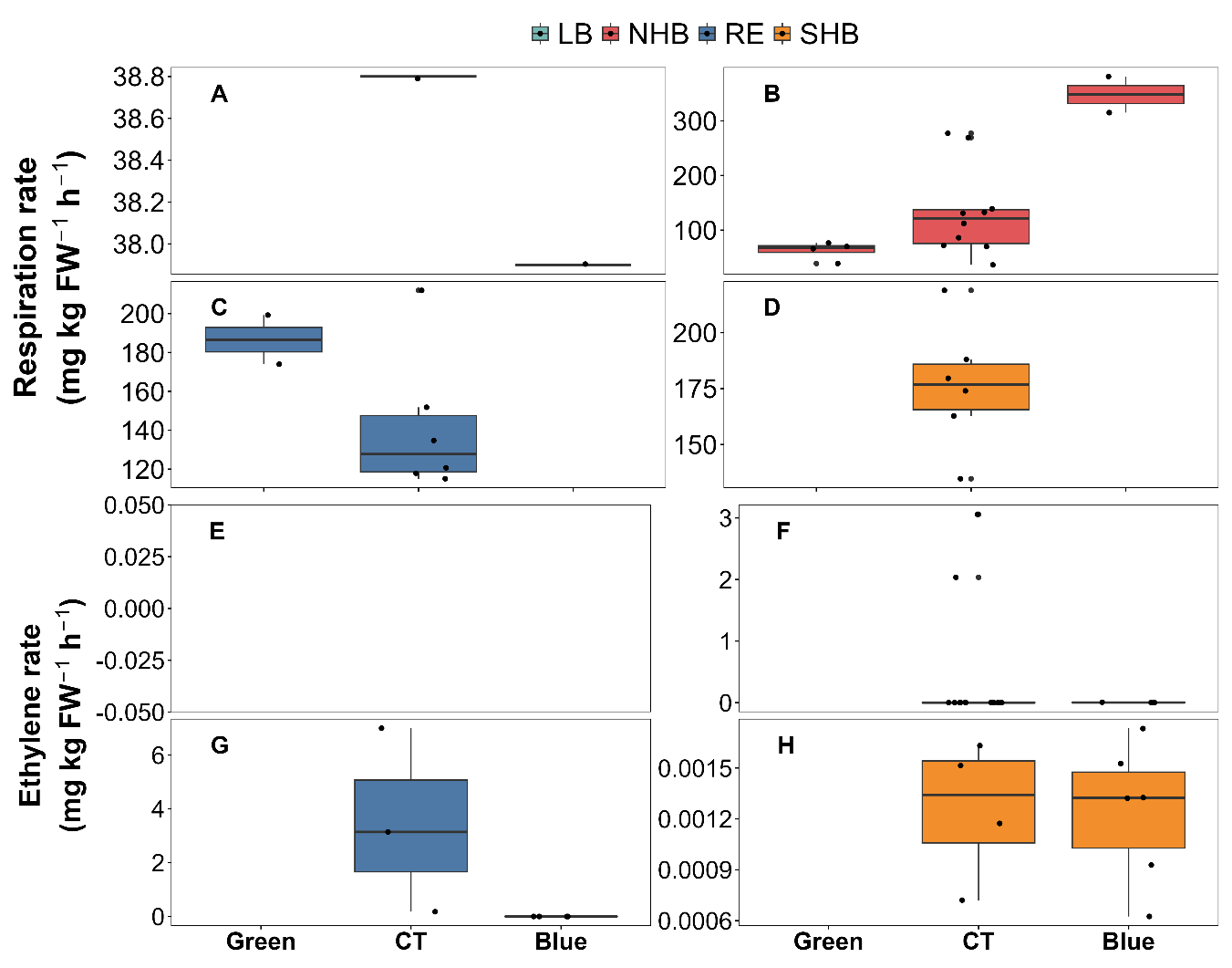


**Supplementary figure 1.** Reported peaks of fruit respiration (CO_2_; A-D) and ethylene production (C_2_H_4_; E-H) across development stages (x-axis); CT: color transition fruit. Blueberry types: lowbush (LB), northern highbush (NHB), rabbiteye (RE) and southern highbush (SHB). Ethylene data for lowbush blueberries (E) were not available in the literature. Values of respiration (*n*= 32) and ethylene (*n*= 35) are expressed in mg kg FW^-1^ h^-1^ to facilitate visualization and analysis.
